# Influence of Cocatalysts (Ni, Co, and Cu) and Synthesis Method on the Photocatalytic Activity of Exfoliated Graphitic Carbon Nitride for Hydrogen Production

**DOI:** 10.3390/nano12224006

**Published:** 2022-11-14

**Authors:** Adeem Ghaffar Rana, Michael Schwarze, Minoo Tasbihi, Xavier Sala, Jordi García-Antón, Mirjana Minceva

**Affiliations:** 1 Biothermodynamics, TUM School of Life Sciences, Technical University of Munich, Maximus-Von-Imhof-Forum 2, 85354 Freising, Germany; 2 Department of Chemical, Polymer and Composite Materials Engineering, University of Engineering and Technology (UET), Lahore 39161, Pakistan; 3 Department of Chemistry, Technische Universität Berlin, Straße des 17. Juni 124, 10623 Berlin, Germany; 4 Departament de Química, Unitat de Química Inorgànica, Universitat Autònoma de Barcelona, 08193 Bellaterra, Barcelona, Spain

**Keywords:** graphitic carbon nitride, water splitting, hydrogen production, nickel, cocatalyst deposition

## Abstract

Exfoliated graphitic carbon nitride (ex-g-CN) was synthesized and loaded with non-noble metals (Ni, Cu, and Co). The synthesized catalysts were tested for hydrogen production using a 300-W Xe lamp equipped with a 395 nm cutoff filter. A noncommercial double-walled quartz-glass reactor irradiated from the side was used with a 1 g/L catalyst in 20 mL of a 10 vol% triethanolamine aqueous solution. For preliminary screening, the metal-loaded ex-g-CN was synthesized using the incipient wetness impregnation method. The highest hydrogen production was observed on the Ni-loaded ex-g-CN, which was selected to assess the impact of the synthesis method on hydrogen production. Ni-loaded ex-g-CN was synthesized using different synthesis methods: incipient wetness impregnation, colloidal deposition, and precipitation deposition. The catalysts were characterized by X-ray powder diffraction, X-ray photoelectron spectroscopy, nitrogen adsorption using the Brunauer–Emmett–Teller method, and transmission electron microscopy. The Ni-loaded ex-g-CN synthesized using the colloidal method performed best with a hydrogen production rate of 43.6 µmol h^−1^ g^−1^. By contrast, the catalysts synthesized using the impregnation and precipitation methods were less active, with 28.2 and 10.1 µmol h^−1^ g^−1^, respectively. The hydrogen production performance of the suspended catalyst (440 µmol m^−2^ g^−1^) showed to be superior to that of the corresponding immobilized catalyst (236 µmol m^−2^ g^−1^).

## 1. Introduction

Energy and environmental challenges are emerging with industrial and economic development [1,2,3]. Industrialization and increasing population are the main reasons behind the energy and environmental crises. Most of the world’s energy demand is fulfilled by nonrenewable sources (petrol, diesel, and coal), which are becoming depleted [4,5]. Additionally, during the past few decades, the increased consumption of fossil fuels has led to severe environmental issues, such as global warming and climate change. As a result, researchers are putting more effort into developing renewable energy sources, the only alternative to ensure sustainable development. Solar light, wind, biomass, hydro, and geothermal energy sources are eco-friendly sources of renewable energy [6,7,8,9]. Among them, sunlight energy can be stored in the chemical bonds of a fuel (e.g., hydrogen, H_2_) through artificial photosynthesis [4,10,11,12]. Hydrogen, an alternative renewable energy source, can release considerable energy without emitting greenhouse gases; water is the only by-product of hydrogen combustion. Hence, photocatalytic H_2_ generation through solar light-driven water splitting using semiconductor materials that convert solar energy to chemical energy has become a promising approach [13]. Consequently, the development of a stable, effective, and inexpensive catalyst for the hydrogen evolution reaction (HER) has become a challenging and important research topic [14].

A wide variety of semiconductor-based photocatalysts, such as TiO_2_ [15], ZnO [16,17], Bi [18], CdS [19], and g-CN [20], have been investigated in the last few decades. The more conventional (i.e., TiO_2_ and ZnO) have advantages and disadvantages regarding stability and nontoxicity [21,22]. Conversely, they typically have large bandgaps and a high rate of electron–hole recombination, which results in low solar-to-fuel efficiency [23]. Among semiconductor materials, graphitic carbon nitride (g-CN) has attracted considerable attention since Wang et al. first reported its use for water splitting [14,24]. g-CN, a visible-light photocatalyst composed of N, C, and H, has attracted interest because of its extensive application in CO_2_ reduction [25], pollutant degradation [23], organic synthesis reactions [26], and water splitting [27]. These nitrogen-rich materials are inexpensive, abundant, and easy to synthesize [22,28]. The thermal decomposition of nitrogen-rich precursors, such as melamine, urea, thiourea, cyanamide, or dicyanamide, is used to synthesize g-CN in the form of tri-s-triazine sheets. Moreover, the nontoxic g-CN, which can be activated by visible light because of the low bandgap energy (2.7 eV), possesses chemical, electronic, and thermodynamic stability [22,23,29]. However, the photocatalytic performance of bulk g-CN is low because of the low specific surface area (10 m^2^/g), low availability of active sites, low adsorption and absorption, and rapid recombination of photogenerated electron-hole pairs. Several strategies can be adopted during the photocatalyst design to improve the surface and optical properties of g-CN to overcome these problems. These include metal and nonmetal doping, morphology control, compositing with other semiconductor materials, and exfoliation [22,30]. From these strategies, exfoliation is a fast, efficient, and easy method to improve a given material and its optical properties by increasing its surface area [23]. Moreover, doping can help improve the electronic properties of materials by introducing more electron-hole pairs and minimizing charge recombination [8,22,24,30].

Modifying the catalyst with a cocatalyst enhances the performance of materials significantly. Immobilization of a cocatalyst on the surface of the semiconductor material is one of the efficient and essential methods to accelerate the separation efficiency of electron–hole pairs generated in the process, consequently enhancing the overall photocatalytic performance of the semiconductor material. Therefore, adding cocatalysts enhances hydrogen production in a water-splitting reaction. Noble and non-noble metals can play this role [1,6,13,21,31].

Noble metal cocatalysts typically enhance the kinetics of the reaction at low overpotentials and help induce charge separation from the semiconductor to the cocatalyst [13]. Noble metals, such as platinum (Pt) [32], gold (Au) [33], ruthenium (Ru) [34], silver (Ag) [35], and palladium (Pd) [36], are usually the most common cocatalysts for improving the photocatalytic performance [1]. However, noble metals are scarce, which prevents their practical implementation on a large scale. Hence, researchers are trying to find a cocatalyst that can replace these noble metals, such as non-noble metals, metal oxides, and metal sulfides [21,31]. From all the non-noble metals available for HER, nickel-based cocatalysts have attracted attention because of their low price, stability, and high activity [1].

Recently, Ni metal and Ni-based compounds (Ni_2_P, Ni(OH)_2_, NiN_3_, NiB, NiS, and Ni_3_C) have shown significant HER performance when used as cocatalysts in g-CN-based photocatalytic systems. Ni can play a similar role to noble metals, improving the separation efficiency of electron–hole pairs [1,13]. The cocatalyst loading on the support material can be achieved using diverse synthetic methods. The synthesis methods determine the structure, dispersion, and size distribution of the cocatalyst, which affects the activity of the final hybrid material. In recent years, various methods have been proposed to synthesize supported nanoparticles. However, few studies have compared different synthesis methods for a given support and a given photocatalytic processes. Therefore, rationally choosing the most suitable method remains a challenge [37].

This study evaluated the effect of non-noble metal (Ni, Cu, and Co) cocatalyst loading on exfoliated g-CN (ex-g-CN) for hydrogen production. The hybrid photocatalyst was initially prepared using the incipient wet impregnation (IWI) method and the superior photocatalytic activity of Ni (over Cu and Co) was observed. After this preliminary screening, the best-performing Ni-based hybrid photocatalyst was prepared using one of the following methodologies: (i) precipitation deposition method (PRDM), (ii) IWI method, and (iii) colloidal deposition method (CM). The effects of the synthesis method on the HER photocatalytic performance were studied with triethanolamine (TEOA) as the sacrificial agent under simulated solar light irradiation using a 300-W xenon lamp with a 395-nm cutoff filter. Moreover, the effects of the catalyst exposure to the reaction medium (immobilization or suspension) were studied for practical applications.

## 2. Materials and Methods

### 2.1. Chemicals

Melamine (C_3_H_6_N_6_, 99%) and dihydrogen hexachloroplatinate(IV) hydrate, 99.9% (metal basis) (H_2_PtCl_6_·H_2_O) were purchased from Alfa Aesar (Haverhill, MA, USA). Nickel sulfate hexahydrate (NiSO_4_·6H_2_O), copper sulfate pentahydrate (CuSO_4_·5H_2_O), and cobalt sulfate monohydrate (CoSO_4_·H₂O) (Merck, Darmstadt, Germany), sodium hydroxide (NaOH), ethanol (C_2_H_5_OH), methanol (CH_3_OH), cyclohexane (C_6_H_12_), 1-butanol (C_4_H_10_O), and ascorbic acid (C_6_H_8_O_6_) were obtained from VWR (Radnor, PA, USA). Triton X-100 (C_14_H_22_O(C_2_H_4_O)_n_ (n = 9–10)) was supplied by Sigma-Aldrich (Darmstadt, Germany). TEOA (C_6_H_15_NO_3_) was acquired from Sigma-Aldrich (Darmstadt, Germany). All chemicals were used as received.

### 2.2. Synthesis of ex-g-CN

Bulk carbon nitride (g-CN) was synthesized with prethermal decomposition of melamine using the procedure established in a previous study [23]. Briefly, a closed crucible with melamine was placed into a muffle furnace (Carbolite Gero, GPC 1200, Derbyshire, UK) for thermal decomposition. The heating program consisted of two steps: heating to 450 °C with a gradient of 2 °C min^−1^, and this temperature was kept for 2 h. The sample was then heated to 550 °C at a rate of 2 °C min^−1^, maintaining this temperature for 4 h. Bulk g-CN was crushed in a mortar and pestle, rinsed with ultrapure water, and dried overnight at 80 °C. Carbon nitride (ex-g-CN) was exfoliated from g-CN by placing g-CN in an open crucible inside a muffle furnace for 2 h at 500 °C using a heating ramp of 2 °C min^−1^. The ex-g-CN was obtained after thermal treatment.

### 2.3. Cocatalyst Loading onto ex-g-CN

#### Incipient Wet Impregnation

The IWI method was used to synthesize Ni, Cu, Co, and Pt-loaded ex-g-CN catalyst (2 wt.% theoretical loadings) for the preliminary screening of hydrogen production. The Ni-based catalyst synthesized using this method was called Ni_IWI_/ex-g-CN [38]. According to stoichiometric calculations, the required amount of a respective salt precursor was added dropwise to the ex-g-CN. The beaker containing the catalyst powder was placed in a sonicator for better dispersion. The material was dried overnight at 80 °C before further use.

### 2.4. Precipitation Deposition Method

Ni_PRDM_/ex-g-CN (2 wt.% theoretical Ni loading) was synthesized using the PRDM [39]. The respective amount of the nickel salt precursor was added to 300 mL of milli-Q water. The solution was maintained at pH 9 using 0.1M NaOH. At a stable pH, ex-g-CN was added with continuous stirring. The deposition–precipitation procedure was conducted at 70 °C at a constant pH for 2 h, and the slurry was stirred and dried overnight at the same temperature. The catalyst was washed and dried at 80 °C under vacuum.

### 2.5. Colloidal Deposition Method

Ni_CM_/ex-g-CN (2 wt.% theoretical Ni loading) was synthesized using the CM. The microemulsion system for synthesizing Ni nanoparticles consists of two phases, e.g., the water and oil phases. The nickel salt precursor, surfactant (Triton X-100), and co-surfactant (1-butanol) were present in the water phase. The oil phase was cyclohexane. Two microemulsions were prepared for the synthesis: (1) with Ni salt and (2) with a reducing agent (ascorbic acid). The synthesis process was conducted in a glass reactor with stirring. Emulsion 2 contained ascorbic acid, cyclohexane, butanol-1, triton X-100, and water. Emulsion 1 contained the nickel salt precursor, cyclohexane, butanol-1, triton X-100, and water. Emulsion 2 was added slowly to the reactor containing emulsion 1. The mixture was stirred at 700 rpm for 30 min at room temperature to form the colloidal stabilized Ni nanoparticles and then stirred for 2 h at room temperature. The ex-g-CN support was then added to the mixture with vigorous stirring to deposit the Ni nanoparticles onto the surface of ex-g-CN. The suspension was stirred further for 2 h at 55 °C. After the process, the reactor was cooled to room temperature. The solid was centrifuged and washed several times with acetone. Finally, the catalyst was dried at 80 °C under vacuum.

### 2.6. Characterization

The Brunauer–Emmett–Teller (BET) surface area was determined from a nitrogen adsorption–desorption experiment at 77 K (quadrasorp, Quantachrome, Boynton Beach, FL, USA). The crystalline phases were examined using X-ray diffraction (XRD, Mini Flex 600C, Rigaku, Tokyo, Japan) was conducted using CuKα radiation at a voltage, current, and spin speed of 40 kV, 15 mA, and 80 rpm, respectively, in the range between 3° and 60° 2θ with a step size of 0.0075°. Transmission electron microscopy (TEM, JEM-ARM300F2) images were obtained using a probe corrected with a cold FEG emitter (JEOL Ltd., Tokyo, Japan) operated at 300 kV with a camera length of 10 cm. The acquired and evaluated high-angle annular dark-field images represent a detection angle of 54–220 mrad. The image contrasts were formed mainly by Rutherford scattering and were correlated to the atomic number. X-ray photoelectron spectroscopy (XPS, Phoibos 150 analyzer, SPECS GmbH, Berlin, Germany) was conducted under ultra-high vacuum conditions (base pressure 5 × 10^−10^ mbar) using a monochromatic Al Kα X-ray source (1486.74 eV). The energy resolution measured from the FWHM of the Ag 3d^5/2^ peak for a sputtered silver foil was 0.62 eV at the Catalan Institute of Nanoscience and Nanotechnology in Barcelona. Ni loading in Ni-loaded ex-g-CN samples was determined using inductively coupled plasma optical emission spectrometry (Perkin-Elmer Optima 4300DV model system) in the Chemical Analyses Service of the Universitat Autònoma de Barcelona.

### 2.7. Photocatalytic Experiments

The photocatalytic experiments were performed in a noncommercial side-irradiated double-walled quartz-glass reactor (shown in a previous work [40]) with a maximum volume of 35 mL. In general, 20 mg of the prepared photocatalyst was placed into the reactor, and 20 mL of an aqueous TEOA solution (10 vol% TEOA) was added. The solution was flushed with Ar for 15 min to remove oxygen, and the reactor was closed with a rubber septum. The reactor was connected to a thermostat (ministat 125, Huber, Germany, T_set_ = 19 °C) and placed onto a magnetic stirrer in front of a 300-W Xe lamp equipped with a 395-nm cutoff filter. The distance between the lamp and reactor was 10 cm. The lamp was switched on, and after irradiating the intensively stirred photocatalyst suspension for the desired time, a gas phase sample was taken with a gas-tight syringe. The gas sample was analyzed by gas chromatography (Agilent Technologies 7890 A) equipped with a thermal conductivity detector.

The hydrogen production photocatalytic activity was calculated as follows:

(1) $$H_{2}\;\left({\mu \mathrm{mol}\;g}^{-1}h^{-1}\right)=\frac{\left(V_{0}-V_{L}\right)\cdot H_{2}\left(GC\right)}{m_{cat}\cdot t_{R}\cdot V_{m}}$$

where *V*_0_ is the total volume of the reactor, *V_L_* is the volume of the solution, *H*_2_ is the amount of *H*_2_ detected using GC, *m_cat_* is the amount (g) of catalyst, *t_R_* is the irradiation time, and *V_m_* is the molar volume of hydrogen.

## 3. Results

### 3.1. Catalyst Characterization

Before assessing the as-prepared photocatalysts for the HER, they were characterized using the different techniques. Optical properties were characterized using UV-Vis and photoluminescence (PL) spectroscopy (Figures S1 and S2). The bandgap energy for bulk and exfoliated g-CN were 2.42 and 2.62 eV showing light absorption in the visible range. After loading with Co, Ni, and Cu, the bandgap energy was 2.6, 2.5, and 2.4 eV, respectively. The optical properties remained after cocatalyst loading. In the case of different methods to load Ni onto ex-g-CN, bandgap energy was constant at about 2.5 eV. PL spectroscopy showed that g-CN materials absorbed the maximum in the range of 430–460 nm. The chemical composition was studied using FTIR (Figure S3) and spectra are typical for g-CN materials. Peaks at 806 cm^−1^ can be attributed to triazine units, whereas the strong bands between 1636 and 1242 cm^−1^ belong to the C=N and C–N bonds of heterocyclic rings. There were no peak alterations and new peaks after the addition of the cocatalyst.

### 3.2. X-ray Diffraction

**Figure 1:**
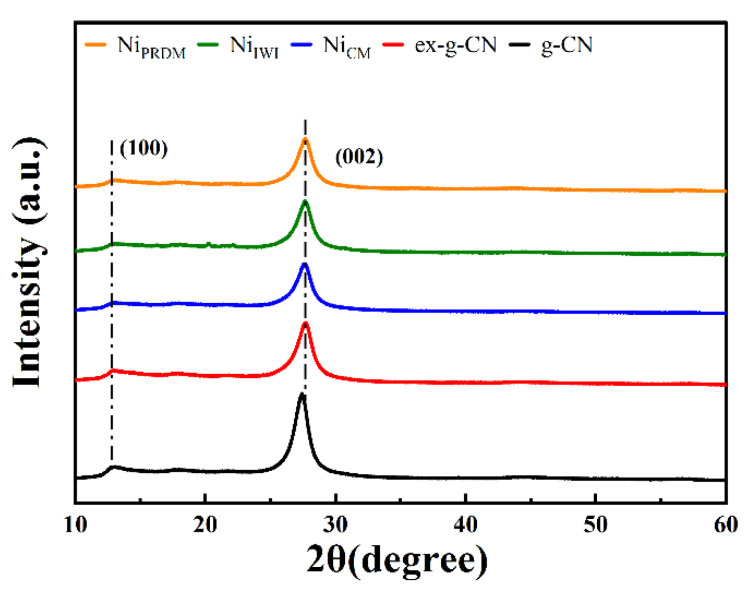
XRD patterns of g-CN, ex-g-CN, and Ni-loaded ex-g-CN synthesized using the incipient wet impregnation (IWI) method, colloidal method (CM), and precipitation deposition method (PRDM).

The phase structures of g-CN, ex-g-CN, and Ni-loaded ex-g-CN were examined using XRD and the diffractograms are shown in Figure 1. All samples showed the characteristic peak at 27.2° 2θ and a weaker peak at 13° 2θ. The strong peak was indexed to the (002) plane, a characteristic interlayer stacking peak of aromatic g-C_3_N_4_ systems. Moreover, the weak peak was assigned to the (100) crystal plane, which was attributed to the repeated tri-s-triazine units. The decrease in intensity and the slight shift in the peaks were attributed to exfoliation and Ni loading. There were no peaks of Ni due to the low Ni content. The XRD patterns of cobalt and copper loaded ex-g-CN showed similar peaks with no visible peak of respective metal (Figure S4).

### 3.3. Brunauer–Emmett–Teller

The specific surface areas were measured to reveal any change in the structural features of carbon nitride before and after exfoliation. The surface area of ex-g-CN, obtained from the N_2_ adsorption-desorption curves (Figure S5), was significantly higher than that of g-CN (169.3 m^2^/g vs. 5.4 m^2^/g, respectively). The surface areas of the Ni-loaded ex-g-CN, Ni_PRDM_, Ni_IWI_, and Ni_CM_ were 84.6, 69.8, and 65 m^2^/g, respectively. The reduction in the surface area is probably due to blockage of the pores with the loaded metal particles.

### 3.4. Inductively Coupled Plasma (ICP) Mass Spectrometry (MS)

The theoretical loading of Ni for all samples was 2%. The Ni content of the Ni_PRDM_, Ni_IWI_, and Ni_CM_, determined using ICP-OES, were 1.7, 1.2, and 1.4 wt.%, respectively, corresponding to Ni deposition yields of 85%, 60%, and 70%, respectively. The Ni loading ratio on the surface of g-ex-CN was high compared with that of Pt. The surface charge of Ni was positive, and g-CN was negative, whereas the surface charge of Pt was negative. Because of the negative charge of Pt, the deposition yield for Pt obtained in earlier investigations was consistently lower (below 50%) [41,42]. The copper and cobalt actual loadings were 1.7% and 1.4%, respectively (theoretical loading in both cases was 2%).

### 3.5. X-ray Photoelectron Spectroscopy

**Figure 2:**
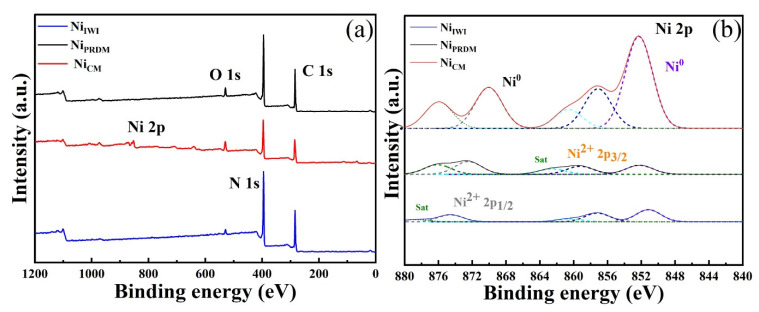
XPS profiles of the obtained samples for (**a**) survey and (**b**) Ni 2p scan.

XPS measurements were conducted to investigate the composition of the Ni-loaded ex-g-CN photocatalysts. As shown in Figure 2, the XPS survey spectra confirmed the presence of C, N, O, and Ni. Ni was visible in the sample prepared using the CM. Figure 2b presents the high-resolution spectra of Ni 2p. Four significant peaks were observed at 852.3, 857.2, 870.1, and 873.5 eV. The peaks at 852.3 and 870.1 eV correspond to metallic Ni. These peaks were clearly visible in the sample prepared using the CM (Ni_CM_), but they were either reduced in intensity (Ni_PRDM_) or absent (Ni_IWI_) for the other two samples, even if Ni was detected using ICP-MS. The other two peaks at 857.2 and 873.1 eV correspond to Ni^2+^. The two peaks at 862.5 and 876.8 eV are satellite peaks of Ni^2+^. Ni always showed strong satellite peaks approximately 6 eV above the main electronic lines [31,43,44,45,46,47]. The XPS peaks of cobalt and copper loaded ex-g-CN (Figure S6) confirmed the presence of these species in different oxidation states.

### 3.6. Morphology

**Figure 3:**
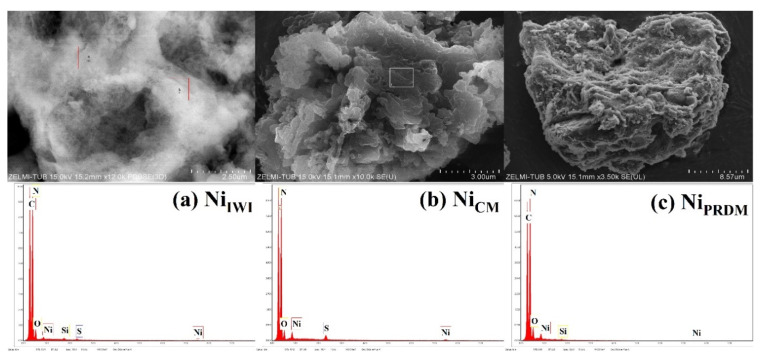
SEM images and EDX spectra of Ni-loaded ex-g-CN synthesized using the (**a**) incipient wet impregnation (IWI) method, (**b**) colloidal method (CM), and (**c**) precipitation deposition method (PRDM).

**Figure 4:**
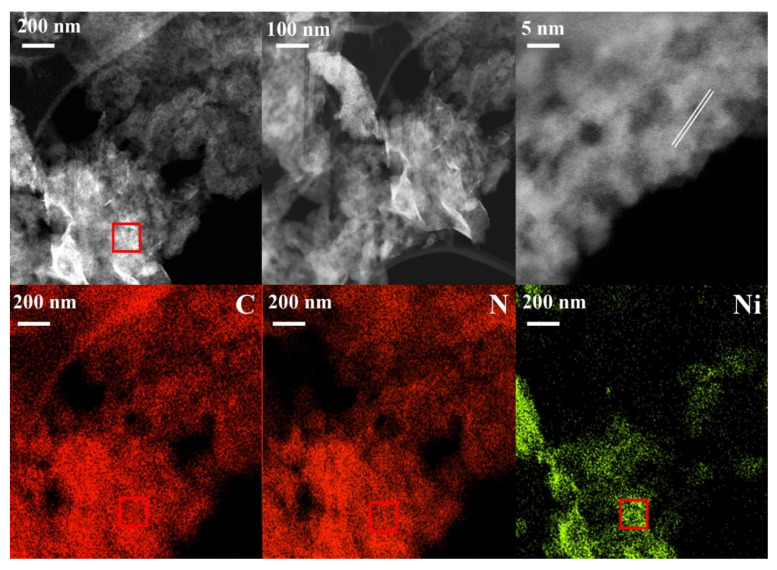
TEM images at different resolution and EDX mapping (from top left image) of Ni_CM_.

The morphology of the Ni-based catalysts synthesized using different methods was investigated using SEM (Figure 3). The catalysts synthesized using the CM were studied further using TEM (Figure 4). All catalysts showed a stacked morphology, and EDX analysis (Figure 3) confirmed the Ni loading. In Figure 4, TEM images of the Ni-based catalyst synthesized using the CM at different magnifications are presented together with the corresponding EDX elemental mapping. A flat-sheet structure with thin layers was observed. EDX mapping showed that the synthesis method yielded Ni species loaded on the ex-g-CN surface. TEM images and EDX spectra of cobalt and copper loaded ex-g-CN are shown in Figure S7. They do not show nanoparticles on the surface.

### 3.7. Photocatalytic H_2_ Production Activity

The photocatalytic activity of the synthesized hybrid materials was studied for hydrogen production. First, the metal-loaded ex-g-CN catalysts synthesized using the IWI method were screened. The best-performing non-noble metal was chosen for further study. Second, the best-performing non-noble metal-based catalyst was synthesized with different methods, and the effects of the synthesis method on H_2_ production were studied. Furthermore, the effects of the catalyst concentration and catalyst exposure to reaction medium (immobilized or suspended) on hydrogen production were studied. The reactions were performed in the presence of TEOA as a sacrificial electron donor, and the HER activities were evaluated under visible-light irradiation (λ > 396 nm).

### 3.8. Screening Experiments

**Figure 5:**
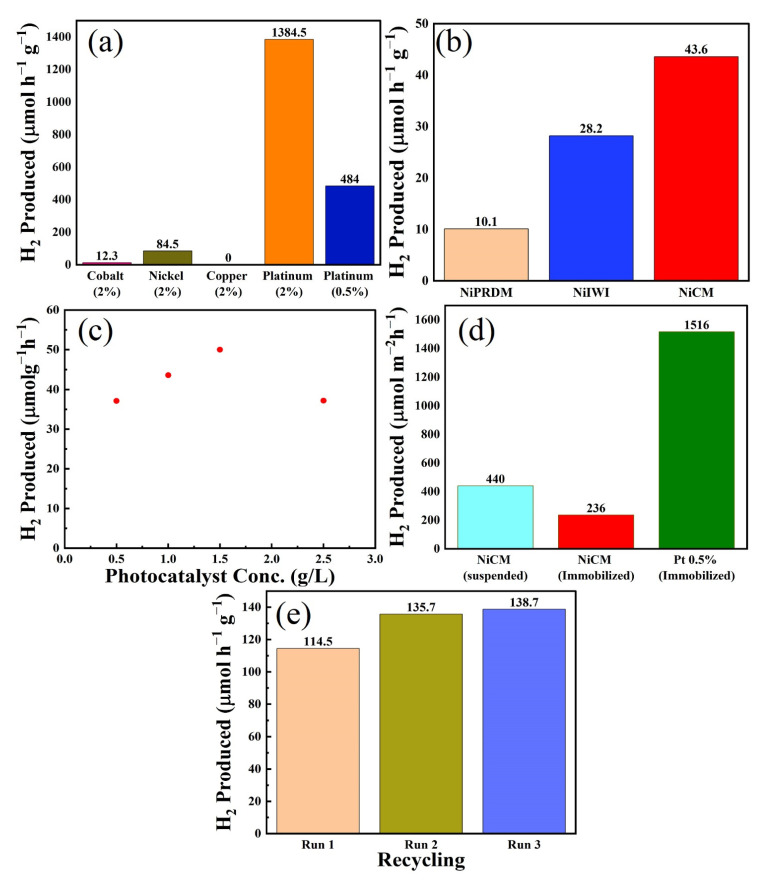
(**a**) Comparison of the photocatalytic H_2_ evolution with different noble and non-noble metals; (**b**) photocatalytic H_2_ production activities of different Ni-based samples under visible-light irradiation for 6 h; (**c)** effect of the catalyst concentration on the hydrogen production; (**d**) photocatalytic H_2_ evolution of suspended and immobilized Ni_CM_-based photocatalysts and analogous immobilized Pt-based photocatalyst; and (**e**) re-utilization of the catalyst (estimated error 10%).

Screening experiments were performed for the synthesized catalysts; the results are presented in Figure 5a. Almost negligible hydrogen was produced for ex-g-CN without a cocatalyst after 6 h [48]. Ni (2% loading) performed best with 84.5 µmol h^−1^ g^−1^ hydrogen produced, followed by 2% loading on cobalt (12.3 µmol h^−1^ g^−1^) and copper (0 µmol h^−1^ g^−1^). The catalyst performed in the following order: Ni > Co > Cu = ex-g-CN [48,49,50]. Pt (2% and 0.5% loading) outperformed all non-noble metal-based photocatalysts for hydrogen production (Figure 5a). Further experiments were performed with the best-performing non-noble metal Ni system.

### 3.9. Effect of the Ni Loading Method on Hydrogen Production

After selecting Ni as a cocatalyst for further investigations, the effects of the loading method on H_2_ production were studied, and the results are presented in Figure 5b. Ni-loaded ex-g-CN synthesized using the CM performed best, with a H_2_ production rate of 43.6 µmol h^−1^ g^−1^. The presence of Ni nanoparticles on the material surface (confirmed by TEM, vide supra) could explain the higher photocatalytic activity of this system. The catalysts synthesized using the impregnation and precipitation methods were less active, with H_2_ production rates of 28.2 and 10.1 µmol h^−1^ g^−1^, respectively. In addition to XPS, although ICP and EDX verified the presence of Ni, no nanoparticles were observed by TEM, which could explain the lower activity of these catalysts. A plausible explanation could be that the Ni precursor is still present on ex-g-CN after synthesis and was reduced to Ni during irradiation (via photodeposition). Further experiments were performed using the best-performing Ni_CM_.

### 3.10. Effect of Photocatalyst Concentration

The effects of photocatalyst concentration on hydrogen production were examined by varying the mass of Ni_CM_ between 10 and 50 mg, keeping the liquid volume (20 mL) constant, resulting in a variation of the photocatalyst concertation between 0.5 and 2.5 g/L. Figure 5c shows the hydrogen produced as a function of photocatalyst concentration. No hydrogen production occurred in the absence of the photocatalyst. Hydrogen production initially increased with increasing photocatalyst concentration but decreased at high photocatalyst concentrations after reaching a maximum of approximately 1.5 g/L. The amount of hydrogen produced was 37.1, 43.6, 50, and 37.2 µmol h^−1^ g^−1^ for 10 (0.5 g/L), 20 (1 g/L), 30 (1.5 g/L), and 50 (2.5 g/L) mg of catalyst, respectively. Hydrogen production increased with increasing amount of photocatalyst because of the availability of more active sites for reaction until 2.5 g/L of catalyst. Further increases in photocatalyst concentration decreased hydrogen production because of the hindered light absorption and scattering.

### 3.11. Immobilized vs. Suspended Photocatalysts

Photocatalyst immobilization is essential for technical applications. Therefore, its influence on photocatalytic H_2_ production was studied. The photocatalyst was immobilized onto a filter paper using the method reported by Schwarze et al. [51], and the hydrogen production from the immobilized and suspended catalysts was compared. The results are presented in Figure 5d and were compared with those for the analogous Pt-based immobilized photocatalyst. The same amount of suspended and immobilized catalysts was used in all cases. The suspended Ni-based photocatalyst (440 µmol m^−2^ h^−1^ hydrogen) performed better than the immobilized Ni-based photocatalyst (236 µmol m^−2^ h^−1^ hydrogen). Immobilization led to an approximately 50% loss of photocatalytic hydrogen production activity. This activity loss was expected because not all particles contribute to the reaction when the photocatalyst particles are immobilized as a thin film. The photocatalyst particles deep in the film layer will not be irradiated. Optimizing the film thickness of the catalyst is the key to improving the activity and later use on a larger scale. Furthermore, the immobilized photocatalyst experiments were conducted with a sunlight simulator equipped with an air mass 1.5 global filter with a lower total intensity. The Pt immobilized catalyst produced 1516 µmol m^−2^ g^−1^ H_2_ for only 0.5% loading. Although Pt clearly outperformed Ni as a cocatalyst when combined with ex-g-CN, the use of Ni provides an alternative non-noble metal-based system with good photocatalytic activity.

### 3.12. Recycling of the Catalyst

The recycling of the suspended Ni_CM_ system was demonstrated by performing three cycles of hydrogen production after subjecting the photocatalyst to washing and drying. The amount of hydrogen produced by the photocatalyst increased slightly for at least three consecutive cycles (Figure 5e). In the colloidal method, a reducing agent was used to reduce the Ni salt precursor to Ni nanoparticles. Some residual Ni salt precursor might be adsorbed and reduced during the first irradiation cycle. As a result, more active centers are available for the second run, leading to a higher activity. This assumption was proven by the third run that showed the same activity as the second run. Therefore, the material remained stable, and the slight activation observed potentially arise from changes in the surface composition of the Ni-based cocatalysts under reductive turnover conditions (i.e., changes in the NiO/Ni ratio).

## 4. Conclusions

Non-noble metals (Ni, Cu, and Co) were evaluated as cocatalysts loaded onto ex-g-CN for hydrogen production. The preliminary study on Ni, Cu, and Co synthesized using the IWI method showed that Ni had seven times higher hydrogen production than Co, whereas Cu had little catalytic activity. The Ni-loaded ex-g-CN synthesized using three different synthesis methods was compared: incipient wetness impregnation, colloidal deposition, and precipitation deposition. The Ni_CM_ performed best because of the high loading of nanoparticles on the surface of ex-g-CN confirmed using TEM and XPS. The morphology, structure, and surface properties of the Ni-loaded ex-g-CN photocatalysts were characterized using TEM, XRD, XPS, BET, and ICP, and tested for hydrogen production using a 300 W xenon lamp equipped with a 395 nm cutoff filter. Hydrogen production increased with increasing photocatalyst concentration up to 1.5 g/L. A further increase in the photocatalyst concentration led to lower activity because of hindrance and light scattering. The suspended catalyst performed better than a filter-paper-immobilized counterpart. However, immobilization can be helpful for scale-up and industrial applications. The reduced light penetration onto the film formed upon immobilization leads to the loss of photocatalytic activity because not all particles contribute to the reaction. Furthermore, Ni-loaded ex-g-CN proved to be a stable catalyst that can be recycled and reused without significant loss of activity.

## Data Availability

The data presented in this study are available on request from the corresponding author.

## Supplementary Materials

The following supporting information can be downloaded at: https://www.mdpi.com/article/10.3390/nano12224006/s1, Figure S1: UV-Vis Spectra of synthesized catalysts; inset tauc plots, Figure S2: PL spectra of g-CN and ex-g-CN, Figure S3: FTIR spectra of all synthesized catalysts, Figure S4: XRD patterns of Co- and Cu-loaded ex-g-CN, Figure S5: Adsorption and desorption curves of g-CN and ex-g-CN; inset particle size distribution, Figure S6: XPS profiles of the obtained samples for (a) Co 2p and (b) Cu 2p, Figure S7: TEM and EDX images of Co and Cu-loaded ex-g-CN. References [23,52,53] are cited in Supplementary Materials.
