# Supplementary material for: Influence of Cocatalysts (Ni, Co, and Cu) and Synthesis Method on the Photocatalytic Activity of Exfoliated Graphitic Carbon Nitride for Hydrogen Production
Source: Nanomaterials (Basel). 2022 Nov 14;12(22):4006. doi: 10.3390/nano12224006 (PMC9697847; doi:10.3390/nano12224006)
Supplement: Supplementary file 1 [file nanomaterials-12-04006-s001.zip › nanomaterials-1839169-supplementary.pdf]

nanomaterials

Supplementary Materials

Influence of Cocatalysts (Ni, Co, and Cu) and Synthesis Method on the Photocatalytic Activity of Exfoliated Graphitic Carbon Nitride for Hydrogen Production

The optical properties of pristine and modified g-CN were studied using UV-Vis (Figure S1) and PL spectroscopy (Figure 2). All materials show absorption in the visible range, and the bandgap of the materials change only slightly with modification. Exfoliation leads to a blue shift in the absorption, and its bandgap increases from 2.42 to 2.61 eV. With the addition of metal co-catalysts, the bandgap changes slightly and remains in the same range, absorbing visible light. In the case of Ni, the deposition process had no influence. The PL spectrum shows maximum absorption wavelengths of 458 nm and 436 nm for g-CN and ex-g-CN, respectively. The blue shift is also confirmed by PL spectroscopy.

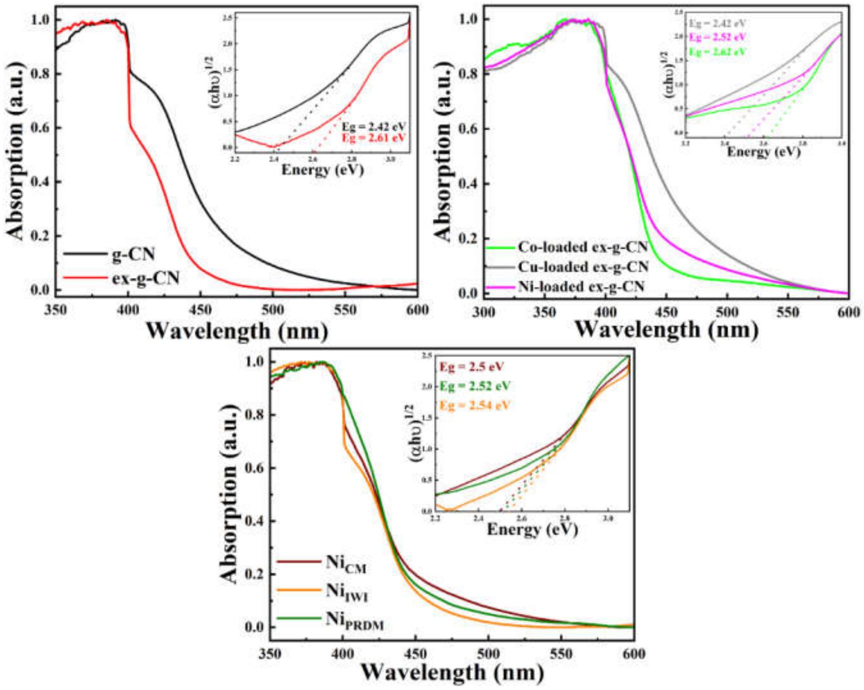

Figure S1. UV-Vis Spectra of synthesized catalysts; inset tauc plots.

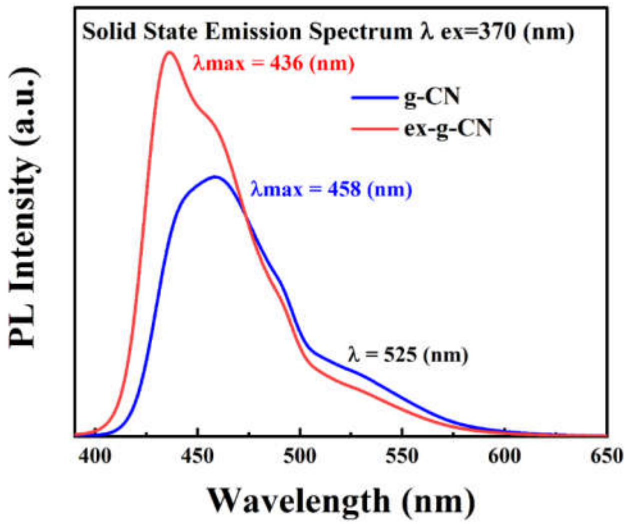

Figure S2. PL spectra of g-CN and ex-g-CN [1].

The chemical composition and the functional groups are studied using FTIR (Figure S3). A broad peak is observed between 3200 and 3000 cm^{-1}, which can be attributed to the stretching vibrations of N–H bonds from residual amino groups and adsorbed H2O. The sharp peak at 806 cm^{-1} can be attributed to the breathing mode of triazine units, whereas the strong bands between 1636 and 1242 cm^{-1} belong to the C=N and C–N bonds of heterocyclic rings. The spectra of all materials show the same absorption bands, the chemical structure remained unaltered after modification, and due to the low content of co-catalysts, there are no peaks of the respective metals.

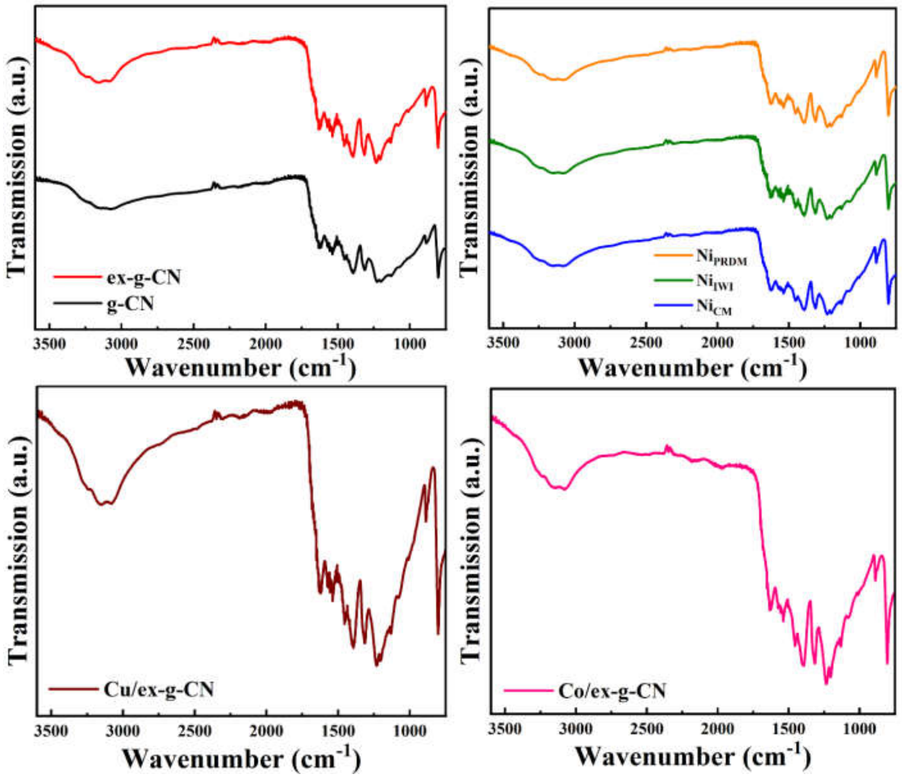

Figure S3. FTIR spectra of all synthesized catalysts.

The XRD patterns of cobalt- and copper-loaded ex-g-CN are shown in Figure S4. It shows typical 2θ peaks of g-C_3N_4 at 13.1° and 27°. Due to the low cocatalyst loading, there are no visible peaks of cobalt or copper.

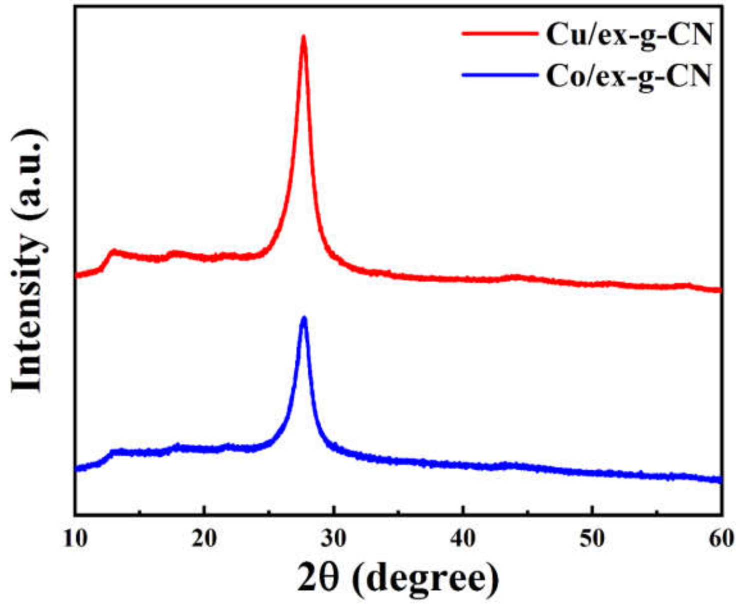

Figure S4. XRD patterns of Co- and Cu-loaded ex-g-CN.

Figure S5 shows the N_2 adsorption-desorption isotherms and BJH pore size distributions of g-CN and ex-g-CN. Both samples show a hysteresis loop of type IV, suggesting the existence of a large number of pores. The pore size distribution curves of both materials show a maximum at a radius of 1.9 nm, which means that the materials were mesoporous [1].

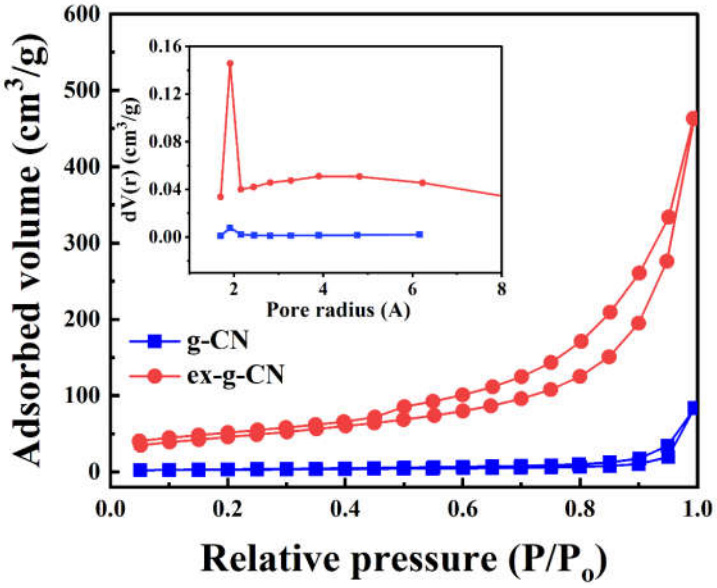

Figure S5. Adsorption and desorption curves of g-CN and ex-g-CN; inset particle size distribution.

The chemical states of cobalt and copper are studied using the XPS and shown in Figure S6. Cobalt is present majorly in oxide or unreduced form in $2p_{3/2}$ and $2p_{1/2}$ states [2]. Copper is also present in the +2 and +1 oxidized states [3].

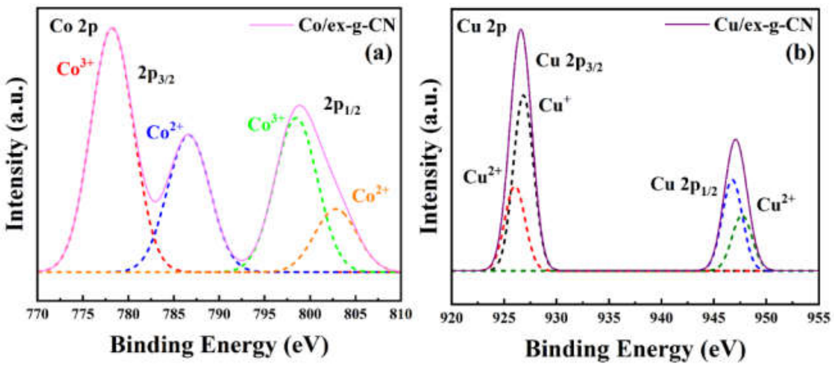

Figure S6. XPS profiles of the obtained samples for (a) Co 2p and (b) Cu 2p.

TEM and EDX of cobalt- and copper-loaded ex-g-CN are shown in Figure S7. EDX confirmed the presence of copper and cobalt on the surface of ex-g-CN. TEM confirmed the nanosheet structure of ex-g-CN, but Co and Cu are not shown in the form of metal nanoparticles.

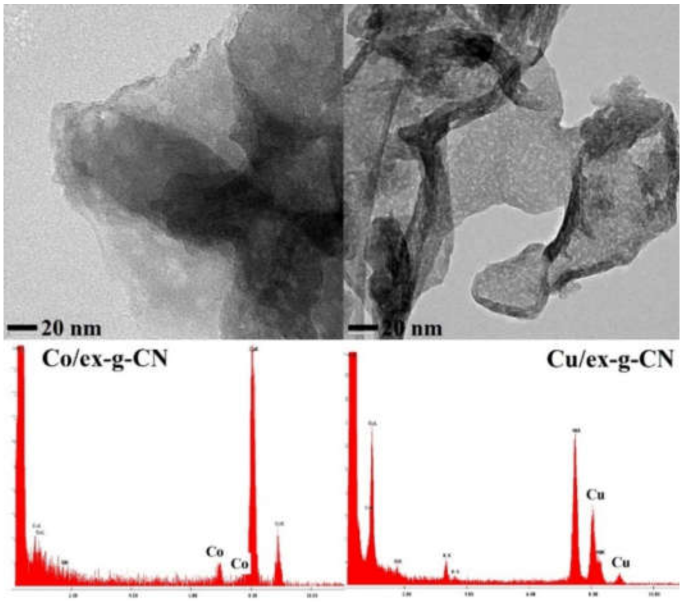

Figure S7. TEM and EDX images of Co and Cu-loaded ex-g-CN.

References

1. Rana, A.; Tasbihi, M.; Schwarze, M.; Minceva, M. Efficient Advanced Oxidation Process (AOP) for Photocatalytic Contaminant Degradation Using Exfoliated Metal-Free Graphitic Carbon Nitride and Visible Light-Emitting Diodes. *Catalysts* **2021**, *11*, 662.
2. Huang, J.; Qian, W.; Ma, H.; Zhang, H.; Ying, W. Highly selective production of heavy hydrocarbons over cobalt–graphene–silica nanocomposite catalysts. *RSC Adv.* **2017**, *7*, 33441–33449.
3. Wang, X.; Zhang, B.; Zhang, W.; Yu, M.; Cui, L.; Cao, X.; Liu, J. Super-light Cu@Ni nanowires/graphene oxide composites for significantly enhanced microwave absorption performance. *Sci. Rep.* **2017**, *7*, 1584.
